# Burnout syndrome in nursing residents in COVID-19 pandemic*

**DOI:** 10.15649/cuidarte.2998

**Published:** 2023-12-20

**Authors:** Stephanie Jully Santos de Oliveira, Wallace Ferreira da Silva, Taís Veronica Cardoso Vernaglia, Silvana Vieira das Chagas, Cristiane Rodrigues da Rocha

**Affiliations:** 1 . Federal University of the State of Rio de Janeiro, Rio de Janeiro, Brazil. Email: stephaniejully70@gmail.com Universidade Federal do Estado do Rio de Janeiro Federal University of the State of Rio de Janeiro Rio de Janeiro Brazil stephaniejully70@gmail.com; 2 . Federal University of the State of Rio de Janeiro, Rio de Janeiro, Brazil. Email: wallacewfs@hotmail.com Universidade do Estado do Rio de Janeiro Federal University of the State of Rio de Janeiro Rio de Janeiro Brazil wallacewfs@hotmail.com; 3 . Federal University of the State of Rio de Janeiro (UNIRIO) IBrazil E-mail: tais.vernaglia@unirio.br Universidade do Estado do Rio de Janeiro Federal University of the State of Rio de Janeiro Brazil tais.vernaglia@unirio.br; 4 . Social Medicine Institute, State University of Rio de Janeiro-UERJ. Brazil Email: silvana@bioestatistica.com Universidade do Estado do Rio de Janeiro Social Medicine Institute State University of Rio de Janeiro Brazil silvana@bioestatistica.com; 5 . Federal University of the State of Rio de Janeiro-UNIRIO. Brazil Email: cristiane.r.rocha@unirio.br Universidade do Estado do Rio de Janeiro Federal University of the State of Rio de Janeiro Brazil cristiane.r.rocha@unirio.br

**Keywords:** Burnout, Psychological, Education, Nursing, Internship and Residency, COVID-19, Agotamiento Psicológico, Educación en Enfermería, Internado y Residencia, COVID-19, Esgotamento Psicológico, Educagao em Enfermagem, Internato e Residencia e COVID-19

## Abstract

**Introduction:**

The incidence of Burnout Syndrome (BS) among nurses is higher. For this group, nursing residents undergo periodic assessments regarding compliance with the care workload in closed sectors, such as Intensive Care and Emergency Care, that put them at higher risk for the syndrome.

**Objective:**

Identify the prevalence and related factors for BS in nursing residents.

**Material and Methods:**

Quantitative, analytical, cross-sectional study with 106 nurses enrolled in the Residency program in Rio de Janeiro, Brazil, based on the following instruments: Maslach Burnout Inventory-General Survey Scale and an occupational sociodemographic questionnaire. Non-parametric statistical tests for independent samples were applied and Mann-Whitney test 15 was used for quantitative variables.

**Results:**

The results show that variable changes in relationships indicates stage 4 of the BS when compared to the other stages. For the other variables, there were no statistically significant differences.

**Discussion:**

Studies reveal that work-family balance is closely related to how job satisfaction is affected and mitigates the negative effects of BS.

**Conclusions:**

Consequences on relation patterns are among the most affected symptoms in the lives of nursing residents. This study is expected to contribute to raising awareness of the problem in question and to improve the quality of work life of resident nurses, detecting and preventing their illness.

## Resumen

**Introducción:**

La incidencia del síndrome de burnout en profesionales de enfermería es mayor. Los residentes de enfermería se someten a evaluaciones periódicas sobre el cumplimiento de la carga de trabajo asistencial en servicios cerrados, como unidades de cuidados intensivos y urgencias, que les exponen a un mayor riesgo de padecer este síndrome.

**Objetivo:**

Identificar la prevalencia y factores relacionados del síndrome de burnout en residentes de enfermería.

**Materiales y métodos:**

Estudio cuantitativo, analítico y transversal que incluyó 106 enfermeros residentes matriculados en el programa de residencia en Río de Janeiro, Brasil, y empleó el Inventario de Burnout de Maslach-Encuesta General y un cuestionario sociodemográfico y ocupacional. Se aplicaron pruebas estadísticas no paramétricas para muestras independientes y se utilizó la prueba de Mann-Whitney para las variables cuantitativas.

**Resultados:**

Los resultados muestran que la variable “cambios en las relaciones” se encuentra en etapa 4 del síndrome de burnout en comparación con las demás etapas. En cuanto a las demás variables, no hubo diferencias estadísticamente significativas.

**Discusión:**

Los estudios revelan que el equilibrio trabajo-familia está estrechamente relacionado con el modo en que se ve afectada la satisfacción laboral y mitiga los efectos negativos del síndrome de burnout.

**Conclusiones:**

Las consecuencias en los patrones de relación se encuentran entre los síntomas más afectados en la vida de los residentes de enfermería. Se espera que este estudio contribuya a concientizar sobre el problema en cuestión y a mejorar la calidad de vida laboral de las enfermeras residentes, detectando y previniendo esta enfermedad.

## Resumo

**Introdujo:**

A incidencia da Síndrome de Burnout (SB) entre os enfermeiros é maior. Para esse grupo, os residentes de enfermagem sao submetidos a avaliagóes periódicas quanto ao cumprimento da carga horária de cuidados em setores fechados, como Terapia Intensiva e Emergencia, o que os coloca em maior risco para a síndrome.

**Objetivo:**

Identificar a prevalencia e os fatores relacionados a SB em residentes de enfermagem.

**Material e métodos:**

Estudo quantitativo, analítico, transversal com 106 enfermeiros matriculados no programa de Residencia no Rio de Janeiro, Brasil, com base nos seguintes instrumentos: Maslach Burnout Inventory-General Survey Scale e um questionário sociodemográfico ocupacional. Foram aplicados testes estatísticos nao paramétricos para amostras independentes e o teste de Mann-Whitney 15 foi usado para variáveis quantitativas.

**Resultados:**

Os resultados mostram que as mudangas variáveis nos relacionamentos indicam o estágio 4 do BS quando comparado com os outros estágios. Para as outras variáveis, nao houve diferengas estatisticamente significativas.

**Discussao:**

Estudos revelam que o equilibrio entre trabalho e família está intimamente relacionado a forma como a satisfagao no trabalho é afetada e atenua os efeitos negativos da SB.

**Conclusóes:**

As consequencias nos padróes de relacionamento estao entre os sintomas mais afetados na vida dos residentes de enfermagem. Espera-se que este estudo contribua para aumentar a conscientizagao sobre o problema em questao e para melhorar a qualidade de vida profissional dos enfermeiros residentes, detectando e prevenindo suas doengas.

## Introduction

Burnout Syndrome (BS) is characterized as a psychological disorder of a depressive character, preceded by intense physical and mental exhaustion caused by exhausting working conditions^1^. This syndrome became a serious health problem specially related to the rising of its epidemic proportion and the notable economic impact^2^. In 2019, BS was included in the 11th Revision of the International Classification of Diseases and declared by The World Health Organization as an occupational phenomenon^3^. Studies reveal a significant rate of this syndrome in health professionals^4^^,^^5^. Several studies reveal that physicians seem to be at risk for burnout BS. A systematic review showed that the prevalence for BS in medical students, physicians in training, and practicing physicians exceeds 50%, which impacts directly in healthcare systems^2^.

For the nurses group, a systematic review that examined the prevalence of the syndrome in 45,539 nurses in 49 countries across multiple specialties showed that one-tenth of the nurses worldwide suffered high burnout symptoms^6^.

For this group, the incidence among nurses is higher, especially those who work in direct contact with patients and their families^1^^,^^4^^,^^7^. The professionals most likely to be affected by the syndrome are those who work in closed sectors, such as Intensive Care and Emergency Care, as well as professionals who develop managerial activities. They have greater physical and psychological overload, which contributes to physical and emotional exhaustion^8^.

Recent research on the prevalence of BS and the associated symptoms identified a significant impact of this syndrome on the working life and quality of life of health professionals, residents, students and teachers^9^^-^^12^.

From these groups students are at greater risk, especially related to years of study. A study that examines the academic burnout of Chinese nursing students in Mainland China, revealed that nursing students of the 4-year undergraduate program had higher academic burnout than students of the 3-year junior program. This outcome may be attributed to the different levels of professional identity and occupational expectation between two education levels nursing students^13^.

Nursing residences students have vulnerable conditions to development BS related to workplace stress, excessive workloads, unemployment, and job insecurity. However there is a lack of knowledge in literature about studies that includes just residency nurse students on the group of investigation. A study that evaluates the impact of anxiety and symptoms of burnout in 752 multi-professional residents in Brazil during the COVID-19 pandemic, included 198 nurses and reveals that nurses present with high levels of symptoms of depression, anxiety, insomnia, and distress^14^. Another study with 78 resident nurses from specialized units of a public university hospital in the city of Rio de Janeiro (Brazil), identified high scores in emotional exhaustion and depersonalization and indicated the need for technical support of the group^15^.

These factors associated with the context of public health emergencies as a result of the outbreak of the new coronavirus (SARS-CoV-2)^10^ place this group in a vulnerable situation for the development of BS. A study that identified the magnitude of the physical and mental effects of BS on health professionals, with an emphasis on comparing the characteristics before and during the COVID-19 pandemic, showed that during the pandemic there was an intensification of symptoms that become constant in everyday life, permeating into a serious state of physical and emotional exhaustion that causes the development and progression of the syndrome over the period^16^^,^^17^. Another study identified that the following are risk factors for the development of Burnout Syndrome in health professionals during the COVID-19 pandemic: work overload, stress, physical exhaustion, depression and compromised social interaction^18^.

Finally, the measures to mitigate the transmission of the virus adopted by educational institutions led to the closure of several establishments and the suspension of several remote face-to-face activities^16^^,^^19^. In this context, residents are facing a change in the ways of teaching and working in the front line to fight this pandemic.

Based on the above, we aim to identify the prevalence and related factors of Burnout Syndrome in Clinical and Surgical nursing residents in the context of the COVID-19 pandemic.

## Material and Methods

### Design and samples

This is a quantitative, cross-sectional, analytical study conducted to identify the prevalence and related factors for BS in 106 nursing residents enrolled in the Residency Program in Clinical and General Surgery, at a public university in Rio de Janeiro, Brazil. On the principle of convenience sampling, the potential sample consisted of 150 nursing residents' students enrolled in the two years of the program. The inclusion criteria were to be regularly enrolled in the first or second year of the Postgraduate Course and to have performed assistance in sectors of assistance to people diagnosed with COVID-19. However, 44 students did not participate based on the inclusion criterio, 15 of the first year and 23 of the second year were in nursing care procedures, 4 were not present in the hospital when the data were collected (may and june/2020), because of sickness and other personal reasons, and 2 leave the Post-Graduation Program.

The nursing residency is a specialization program that takes place over 24 months, with 80% of its workload allocated to practical activities. To complete the course, nursing residents undergo periodic assessments regarding compliance with the care workload (60 hours per week) and must complete theoretical courses and carry out research throughout the program^12^.

### Description of Hospital Units

A total of nine hospital units comprised this study. Two national reference military units for medium and high complexity, elective and emergency procedures. Five federal units that perform major surgeries, specialized and highly complex services. Two specialized units, one reference in the treatment of highly complex heart diseases, and the other specialized in elective surgical care in the field of orthopedics and traumatology.

### Data collection procedures

This study obtained institutional authorization before its beginning. Institutional protocols for carrying out this study were followed. All participants have given informed consent to participate in the research. This research was approved by the Ethics Committee of the educational institution where data collection was performed (CCAE: 27198619.2.0000.5285; Technical Opinion 3.839.084).

The data collection was conducted by two trainees' investigators, supervised by two professors from PhD programs that gave technical assistance and supervision during the fieldwork. For data collection, in order to obtain authorization and support for the application of the instruments we follow the steps described below.

Firstly, contact was made with the preceptorship of each training unit of residents to present the purpose of the study. Secondly, the participants were informed of the study's significance and purpose before giving their express consent. After consent, thirty-minute intervals were used for each resident group, the questionnaire was applied with the purpose of surveying the variables. Finally, all the questionnaires were taken back, governed by confidentiality independence, guaranteeing anonymity of the reporters and security in verification. It is important to note that data collection was carried out into the hospitals during the COVID-19 pandemic period and social distancing and all recommendations from the World Health Organization were respected.

### Data analysis

The database was stored on Mendeley Data^20^. The questionnaires were entered into Microsoft Excel 2010 program with the coding variables to be analyzed. Data processing was made by R 4.0.2 (statistical programming language software). A univariate analysis was conducted in order to describe the profile of the patients who participated in the study. Then, non-parametric statistical tests for independent samples were applied to verify if there is significant evidence of association between the variables of interest. In addition, to verify whether there is evidence of a significant association between the variable “Year” and the Burnout scale score, the Mann-Whitney test was applied, which is applied when there is a categorical variable (Year) and another quantitative variable (Burnout scale score, which was obtained by adding the scale items). Finally, Fisher's exact test was applied, in order to verify whether there is an association between the stages of Burnout and the respective variables of interest. For both statistical methods, the significance level was 0.05^21^^,^^22^.

## Results

The sample consisted of 106 resident nurses from different specialties, of whom 54.71% (n=58) were from the first year (R1) and 45.28% (n=48) from the second year (R2), mostly inserted in activities of general and surgery nursing practice 72.6% (n=77).

To avoid common method bias we ensure that all participants had the necessary experience with the issues of interest, we collected real-world data from only residence nurses, and we collected data only from those respondents who indicated they provided assistance in units for medium and high complexity, elective and emergency procedures, during COVID-19 pandemic.

Also, to notice that despide the different units all of them provided nursing care for medium and high complexity, elective and emergency procedures. The nursing residents were young adults, up to 25 years old, only 3 residents over 40 years old, predominantly female 89.62% (n=95), without stable partners 72.64% (n=77) and belonging to some religion 90.57% (n=96). As for family income, 74.53% (n=79) of the interviewed stated their gross income between 01 (one) and 06 (six) minimum wages.

Regarding the health profile of the participants, a few residents 12.26% (n=13) indicated to undergo psychological counseling, from this group about 1/3 reported having started treatment after the beginning of the residency program. Precisely 18.87% (n=20) reported having increased alcohol consumption after starting their residency program. We also identified that 60.38% (n=64) of nursing residents reported having suffered changes/impacts in their interpersonal relationships after starting the residency program, among which the family member was the most affected. (Table 1).

**Table 1:** Distribution of sociodemographic, occupational and health profile

Characteristic	%(n) (106)
Year	
First year (R1)	54.71(58)
Second year (R2)	45.28(48)
Specialties	
Cardiology	15.09(16)
General practice	72.64(77)
Ortophedics	8.49(9)
Pediatrics	3.77(4)
Martital State. Without stable partner	72.64(77)
Age	
until 25	38.68(41)
26-30	41.51(44)
Gender, Female	89.62(95)
Color auto-declared, White	47.17(50)
Religion	90.57(96)
Psychological counseling	12.26(13)
Increase alcohol consumption	18.87(20)
Interest in emotional support activity at work	77.36(82)
Change in interpersonal relationships	60.38(64)
Lives in the training unit housing, n = 97	40.21(39)

In this research, it was shown that second-year residents have a higher median score for BS when compared to first-year residents. From the hypothesis test, it is observed that there is evidence that there are significant differences between the median of the total score of the syndrome scale between the two groups (47 (40.54); 53 (43.60); p 0.035). For this group, second-year residents (IQR 53) have a higher median score for Burnout syndrome compared to first-year residents (IQR 47) (Table 2).

**Table 2:** Score baseline for Maslach variables

Variables	Total (106)	Year R1 (58)	Year R2 (48)	p-value^1^
Sum of the score				0.035
Average ± SD	49.7 ± 12.27	47.4 (10.942)	52.5 (13.308)	
Median (IQR)	48 (41. 58)	47 (40. 54)	53 (43. 60)	
Amplitude	20. 95	20. 75	22. 95	

1 *Statistical tests performed: U Mann-Whitney Test*

For better understanding and presentation of the multivariate analysis, the ranges of development of Burnout Syndrome were staged from one (1) to five (5). It is noticed a variable change in relationships, where 76.47% of residents are in stage 4 of the syndrome when compared to the other stages. For the other variables, there were no statistically significant differences (Table 3).

**Table 3:** Score baseline for Maslach variables according to sociodemographic, occupational and health profile

Variables	BURNOUT SCALE						
	%(n) (106)	No evidence of Burnout Syndrome (Stage 1) (1)	Possibility to develop (Stage 2) (19)	Threshold of Burnout Syndrome (Stage 3) (68)	Early Burnout Syndrome (Stage 4) (17)	Burnout Syndrome initiated (Stage 5) (1)	p-value^1^
Specialty							0.850
Cardiology	15.09 (16)	0	10.53 (2)	16.18 (11)	17.65 (3)	0	
General Practice	72.64 (77)	100.00 (1)	73.68 (14)	70.59 (48)	76.47 (13)	100.00 (1)	
Orthopedics	8.49 (9)	0	15.79 (3)	8.82 (6)	0	0	
Pediatrics	3.77 (4)	0	0.00 (0)	4.41 (3)	5.88 (1)	0	
Marital Stage							0.289
With stable partner	27.36 (29)	0	42.11 (8)	27.94 (19)	11.76 (2)	0	
Without stable partner	72.64 (77)	100.00 (1)	57.89 (11)	72.06 (49)	88.24 (15)	100.00 (1)	
Religion	90.57 (96)	100.00 (1)	94.74 (18)	92.65 (63)	76.47 (13)	100.00 (1)	0.280
Psychological counseling	12.26 (13)	0	5.26 (1)	13.24 (9)	17.65 (3)	0	0.621
Alcohol consumption	18.87 (20)	100.00 (1)	10.53 (2)	20.59 (14)	17.65 (3)	0	0.342
Interest in emotional support activity at work	77.36 (82)	100.00 (1)	63.16 (12)	80.88 (55)	76.47 (13)	100.00 (1)	0.508
Change in interpersonal relationships	60.38 (64)	0	36.84 (7)	64.71 (44)	76.47 (13)	0	0.022
Lives in the training unit housing	40.21 (39)	0	31.25 (5)	41.94 (26)	47.06 (8)	0	0.852

1 *Statistical tests performed: Fisher's exact test*

## Discussion

The occupations that are focused on caring for the human being are more susceptible to Burnout Syndrome. Among them nursing stands out, which has as characteristics the long working hours, excessive workload and conflicts with the team, patient and family members^1^^,^^4^^,^^5^^,^^7^^,^^12^.

In the context of this research, nursing residents must complete around 2500 hours of activities, sixty hours per week, developed in health units over the 24 months of the course. In addition, residents perform nursing care in different units, developing highly complex, clinical and surgical units, which requires the acquisition of vast theoretical content and practical competence. Studies suggest that BS is high on critical care units when compared to others. For example, a study conducted with more than twenty thousand responses about the relationship between BS and care units, concluded that BS is high among critical care nurses compared to noncritical care^23^.

The workload, context for the participants of this study, can be a predictor for symptoms related to burnout syndrome. Authors review that nurses' workload limited the time that nurses could dedicate to patient care and was related to burnout, also to decreased safety and quality of care, as well as deterioration of job satisfaction^24^.

In addition, the accumulation of tasks related to meeting theoretical and practical requirements can lead to emotional and mental exhaustion. Authors agree that the excessive tasks delegated to the professional can provoke a state of exhaustion, not only physical, but also emotional, specially in the Brazilian scenarios of care^25^.

It is also worth mentioning the gender variable. It is known that nursing is an occupation predominantly composed of women, which is in line with the profile of subjects presented in this research. Studies show that there is a statistically significant relation between the female gender and the symptoms of depersonalization present in the burnout syndrome^26^. Also, in groups of teachers, health professionals and students, higher levels of stress and emotional exhaustion were identified in women when compared to men^27^^-^^29^.

Regarding marital status, it was found that the sample reported not having a stable partner. However, despite for our sample this was not a factor related to the score baseline for BS, different studies reveal that living alone or with just one other person is considered a risk factor for the development of BS. A systematic review that determined the prevalence, levels, and factors related to the burnout syndrome concluded that living alone constitutes the main related factors BS^30^. Other studies indicate that having a stable partner is a protective factor for the development of the syndrome, as the professional would have someone to share the problems of both life and the work environment^10^^,^^31^.

It is noticed that regarding the health profile, the residents reported the need to seek psychological help and suffered changes/impacts in their interpersonal relationships after the beginning of the program. The evidence of the correlation with the BS scale and changes in interpersonal relationship, especially the threshold and early of BS is discussed for other authors. Author points out that work family balance is closely related to how job satisfaction is affected and mitigates the negative effects of BS. For the other hand, work family conflict is considered a predictor for BS^32^. It is known that seeking help is an important strategy to deal with this syndrome, and early identification of associated symptoms is essential^17^.

It can be seen that the change in affective relationships was the variable that indicated a close relationship with BS when compared to the other stages. Studies reveal that being married and having children is a predominant profile in nurses with burnout syndrome. In this way, changes in affective relationships can compromise their support network, an important way of coping with the stressors related to this syndrome^33^.

A possible associated justification that can interfere with distancing and changing family relationships is the fact that almost half of the sample chose to live inhospital accommodation for residents and staff. The fact that residents chose to live inhospital accommodation may be related to the difficulty of getting around and the precarious urban traffic in a big city such as Rio de Janeiro, where the hospital units are located^31^. The increase in traffic problems in large cities and even in the scenario of greater influx of women to the labor market^34^. However, the reduction in travel time between home and work could be a protective factor for BS, and can reduce irritation and fatigue, which compromise development at work^35^.

Residency course seems to be linked to triggering factors of this syndrome. Studies reveal that the factors may be associated with work overload, lack of autonomy, lack of material resources, accumulation of tasks, devaluation and low wages, cause demotivation and personal dissatisfaction, and result in occupational stress, which are directly associated with Burnout Syndrome^10^^,^^11^^,^^17^^,^^18^^,^^31^. However, as far as we know, there is no evidence in literature of a study that compares two different years of nursing residence^36^.

Although this study did not demonstrate the alcohol consumption as a factor related to BS, other authors found out that high exposure to stressors related to the occupation causes pathological responses, such as irritability, and is associated with an increased risk of alcohol consumption^11^^,^^31^^,^^37^.

In this sample, attention is drawn to the fact that residents indicated that they have increased their consumption of alcoholic beverages after starting their residency. In relation to this aspect, it is important to note that there are few studies that address the relation between the pattern of substance consumption and the risk for burnout syndrome^28^^,^^38^. And it was not confirmed Score baseline for Maslach variables according to the sociodemographic profile.

Finally, the sample of residents was restricted to only one higher education institution, and it was a limitation to this study. We chose to apply only the Maslach assessment scale to identify BS. We understand that this scale, when associated with others, could show more explanatory data for the onset of this syndrome. Finally, this is a self-reported survey.

## Conclusions

It is concluded that answered the guide question the changes in relationships indicates a trigger of the BS and consequences on relation patterns are among the most affected symptoms in the lives of nursing residents. Althouth, it is pointed out the work-family balance is closely related to how job satisfaction is affected and mitigates the negative effects of BS.

The results reveal that a large percentage of the interviewed residents are on the threshold for the development of BS, but there was a low occurrence of the initiated syndrome. It is important to highlight that the residents increased their alcohol consumption pattern after the beginning of the course. However, it was not confirmed Score baseline for Maslach variables according to the sociodemographic profile. Therefore, it was not possible to establish a causal link between these variables.It is necessary to better investigate the stressors, which are mostly the long working hours in care, associated with research and teaching, causing problems in the professional and personal areas, which gives rise to personal dissatisfaction associated with professional dissatisfaction due to the lack of appreciation of the occupation and lack of autonomy of the resident, for being a graduate student.

This study is expected to contribute to raising awareness of the problem in question and to improve the quality of work life of resident nurses, detecting and preventing their illness.

In view of the above, it is necessary for institutions to pay attention to the mental health of these residents, outlining preventive actions in order to minimize factors, such as work overload, which contribute to the development of the syndrome.
